# Highly Diverse Arenaviruses in Neotropical Bats, Brazil

**DOI:** 10.3201/eid2812.220980

**Published:** 2022-12

**Authors:** Luiz Gustavo Bentim Góes, Carlo Fischer, Angélica Cristine Almeida Campos, Cristiano de Carvalho, Andrés Moreira-Soto, Guilherme Ambar, Adriana Ruckert da Rosa, Debora Cardoso de Oliveira, Wendy Karen Jo, Ariovaldo P. Cruz-Neto, Wagner André Pedro, Luzia Helena Queiroz, Paola Minoprio, Edison L. Durigon, Jan Felix Drexler

**Affiliations:** Charité-Universitätsmedizin Berlin, Freie Universität Berlin and Humboldt-Universität zu Berlin, Berlin, Germany (L.G.B. Góes, C. Fischer, A.C.A. Campos, A. Moreira-Soto; W.K. Jo, J.F. Drexler);; Scientific Platform Pasteur-USP, São Paulo, Brazil (L.G.B. Góes, A.C.A. Campos, P. Minoprio);; Universidade de São Paulo, São Paulo, Brazil (L.G.B. Góes, A.C.A. Campos, E.L. Durigon);; Universidade Estadual Paulista, Araçatuba, Brazil (C. de Carvalho, W.A. Pedro, L.H. Queiroz);; Universidade Estadual Paulista, Rio Claro, Brazil (G. Ambar, A.P. Cruz-Neto);; Centro de Controle de Zoonooses, São Paulo (A. Ruckert da Rosa, D. Cardoso de Oliveira);; German Centre for Infection Research (DZIF), Berlin (W.K. Jo, J.F. Drexler)

**Keywords:** arenaviruses, Viruses, zoonoses, vector-borne infections, Tacaribe virus, bats, hemorrhagic fever, mammarenavirus, *Tietê mammarenavirus*, Brazil

## Abstract

We detected arenavirus RNA in 1.6% of 1,047 bats in Brazil that were sampled during 2007–2011. We identified Tacaribe virus in 2 *Artibeus* sp. bats and a new arenavirus species in *Carollia* *perspicillata* bats that we named *Tietê mammarenavirus*. Our results suggest that bats are an underrecognized arenavirus reservoir.

Bats are prominent hosts of zoonotic RNA viruses because of immunologic, physiologic, and ecologic factors (*1*). The Arenaviridae family comprises 4 genera: *Reptarenavirus* and *Hartmanivirus*, whose members infect reptiles; *Antennavirus*, whose members infect fish; and *Mammarenavirus*, whose members infect mammals. Mammarenaviruses can be separated into globally distributed lymphocytic choriomeningitis–Lassa virus serocomplex and New World arenaviruses (NWAs) (*2*). The NWAs Junin, Machupo, Sabia, Chapare, and Guanarito cause viral hemorrhagic fever and must be handled under Biosafety Level 4 conditions (*2*). 

All highly pathogenic arenaviruses known thus far are hosted by and transmitted to humans from persistently infected rodents (*2*). Only Tacaribe virus (TCRV; *Tacaribe mammarenavirus*) has been identified in bats (*3*,*4*). Although TCRV is not considered a human pathogen, anecdotal evidence exists for potential laboratory acquired infection that causes influenza-like symptoms (*5*,*6*). In addition, TCRV is phylogenetically related to pathogenic arenaviruses that cause viral hemorrhagic fever; viral properties associated with severe disease, such as evasion of immune responses and cellular tropism, might be conserved in TCRV and genetically related animal arenaviruses (*7*).

Associations between TCRV and *Artibeus* spp. bats are supported only by limited epidemiologic data, including a single virus isolation and serologic evidence (*3*,*4*), considerable illness of bats during experimental infection (*5*), and isolation of TCRV from mosquitoes and ticks that primarily feed on rodents and rarely on bats (*3*,*6*). Limited genetic data exist for TCRV; a single genomic sequence was obtained from a bat-derived isolate generated in the 1950s from Trinidad that has been extensively passaged in mice and cell cultures and another from a recent tick-derived isolate (*3*,*4*,*8*).

## The Study

**Figure 1:**
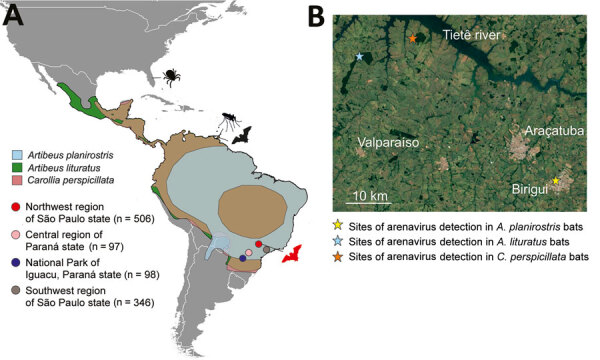
Bat mammarenavirus detection and host distribution in study of highly diverse arenaviruses in neotropical bats, Brazil. A) Geographic ranges of arenavirus-positive bat species indicated by blue (*Artibeus planirostris*), green (*A. lituratus*), and red (*Carollia perspicillata*) colors, according to the International Union for Conservation of Nature (https://www.iucnredlist.org). The brown areas in the map indicate the overlap of the distribution of *A. lituratus* and *C. perspicillata*. The absence of *A. planirostris* distribution in central Brazil likely represents lack of information regarding this species. Filled circles represent regions of sample collection: northwestern region of São Paulo state (red), central region of Paraná state (pink), National Park of Iguaçu, Paraná state (dark blue), and southwestern region of São Paulo state (gray). Number of bats obtained from each region is indicated. Red bat figure indicates where *Tacaribe mammarenavirus* and *Tietê* *mammarenavirus* were detected in the present study. Hosts from which Tacaribe virus was sequenced in other studies, including ticks (Florida, USA), mosquitoes, and bats (Port of Spain, Trinidad and Tobago) are indicated by black pictograms. Map prepared using QGIS desktop software version 3.24 (https://www.qgis.org). B) Areas of arenavirus detection in the northwestern region of São Paulo state, Brazil. Yellow star indicates the capture site of arenavirus-positive *A. planirostris*, blue star indicates the capture site of arenavirus-positive *A. lituratus*, and orange star marks the capture site of arenavirus-positive *C. perspicillata* bats. Tietê River and cities Araçatuba, Valparaíso, and Birigui are indicated. Dark green areas show forest fragments. Map obtained from Google Earth (https://earth.google.com)

We investigated diverse specimens from 1,047 adult bats belonging to 32 species collected from southeastern Brazil (Appendix). We analyzed a total of 3,670 different tissue specimens, including spleens (n = 893), lungs (n = 889), intestines (n = 973), and livers (n = 915), for arenavirus RNA by using reverse transcription PCR (RT-PCR) (*9*) modified to promote NWA amplification (Appendix Table 1, Figure 1).

**Table 1:** Bat species screened for arenaviruses in study of highly diverse arenaviruses in neotropical bats, Brazil

Bat species	Family	No. bats	No. positive (%, 95% CI)*	Region†	Sampling year (no. bats)
*Artibeus fimbriatus*	Phyllostomidae	3	0	A	2012 (3)
*A. lituratus*	Phyllostomidae	155	4 (2.6, 0.7–6.5)	A–D	2007 (8), 2010 (26), 2011 (46), 2012 (45), 2013 (4), 2014 (12), 2015 (16)
*A. obscurus*	Phyllostomidae	2	0	C, D	2013, 2015
*A. planirostris*	Phyllostomidae	9	1 (11.1, 0.3–48.3)	A–C	2010 (3), 2011 (2), 2012 (2), 2013 (1), 2014 (1)
*Carollia perspicillata*	Phyllostomidae	63	12 (19.1, 10.3–30.9)	A–D	2007 (18), 2010 (13), 2011 (18), 2012 (12), 2015 (2)
*Chrotopterus auritus*	Phyllostomidae	1	0	A	2010
*Cynomops planirostris*	Molossidae	11	0	C, D	2013 (1), 2014 (7), 2015 (3)
*Desmodus rotundus*	Phyllostomidae	69	0	C, D	2007 (7), 2011 (44), 2012 (1), 2014 (15), 2015 (2)
*Eptesicus furinalis*	Vespertilionidae	17	0	C, D	2011 (2), 2013 (6), 2014 (3), 2015 (6)
*Eumops auripendulus*	Molossidae	2	0	D	2014, 2015
*E. glaucinus*	Molossidae	106	0	C, D	2009 (1), 2010 (1), 2011 (5), 2012 (5), 2013 (19), 2014 (34), 2015 (41)
*E. perotis*	Molossidae	12	0	C, D	2013 (1), 2014 (8), 2015 (3)
*Glossophaga soricina*	Phyllostomidae	70	0	C, D	2007 (3), 2011 (2), 2012 (1), 2013 (2), 2014 (30), 2015 (32)
*Lasiurus blossevillii*	Vespertilionidae	2	0	C, D	2011, 2012
*L. cinereus*	Vespertilionidae	1	0	C	2013
*L. ega*	Vespertilionidae	2	0	C, D	2013, 2014
*Molossops neglectus*	Molossidae	1	0	D	2014
*M. temminckii*	Molossidae	2	0	C	2011
*Molossus molossus*	Molossidae	242	0	C, D	2007 (1), 2010 (1), 2011 (25), 2012 (16), 2013 (60), 2014 (84), 2015 (55)
*M. rufus*	Molossidae	160	0	C, D	2009 (11), 2010 (1), 2011 (20), 2012 (28), 2013 (48), 2014 (27), 2015 (25)
*Myotis nigricans*	Vespertilionidae	35	0	C, D	2011 (1), 2012 (4), 2013 (9), 2014 (6), 2015 (15)
*M. riparius*	Vespertilionidae	1	0	C	2013
*Noctilio albiventris*	Noctilionidae	2	0	C	2007 (2)
*Nyctinomops laticaudatus*	Molossidae	4	0	C, D	2011 (1), 2014 (2), 2015 (1)
*N. macrotis*	Molossidae	1	0	D	2014 (1)
*Phyllostomus discolor*	Phyllostomidae	2	0	D	2014 (2)
*Platyrrhinus lineatus*	Phyllostomidae	6	0	C, D	2014 (5), 2015 (1)
*Promops nasutus*	Molossidae	1	0	D	2014 (1)
*Pygoderma bilabiatum*	Phyllostomidae	1	0	D	2015 (1)
*Sturnira lilium*	Phyllostomidae	29	0	A, B, D	2010 (5), 2011 (9), 2012 (14), 2015 (1)
*Tadarida brasiliensis*	Molossidae	30	0	C, D	2014 (15), 2015 (15)
*Vampyressa pusila*	Phyllostomidae	1	0	B	2012 (1)
Not identified		4	0	C, D	2011(1), 2013 (2), 2014 (1)
Total	4	1,047	17 (1.6, 0.9–2.6)	A–D	2007–2015

*Number of bats with arenavirus RNA detected by PCR. †Bats were collected from 36 sites within 4 main geographic regions of Brazil: A, Iguaçu National Park; B, central region of Parana state; C, northwest São Paulo state; and D, southwest São Paulo state.

We detected arenavirus RNA in 4 *Artibeus lituratus*, 1 *A. planirostris*, and 12 *Carollia perspicillata* bats; the overall detection rate was 1.62% (95% CI 0.95%–2.59%). Arenavirus-positive bats were collected during 2007–2011 from 3 sampling sites located in both forest and urban areas within a 60-km radius (Figure 1), suggesting arenavirus maintenance in bat populations in this region. All 3 arenavirus-positive bat species are abundant in tropical environments and well-adapted to urban landscapes, indicating potential for dispersion and spillover to humans and other animals.

Most arenavirus-positive bats were collected in 2 forest fragments in 2007 (Tables 1, 2; Figure 1), where most bat species positive for arenavirus RNA were sampled. Whether high detection rates at those sites correspond to epizootics or sampling bias remains unknown.

**Table 2:** Collection sites and arenavirus RNA concentrations in different organs from bats in study of highly diverse arenaviruses in neotropical bats, Brazil*

Sample no.	Bat species†	Sex	Collection site	No. RNA copies/mg tissue				
				Spleen	Lung	Intestine	Liver	Kidney
Br56	*Artibeus lituratus*	M	Valparaiso	5.54 × 10^2^	7.24 × 10^2^	NA	NA	NA
Br57	*A. lituratus*	M	Valparaiso	NA	**3.14 × 10^2^**	NA	NA	NA
Br58	*A. lituratus*	M	Valparaiso	**2.15 × 10^6^**	**6.13 × 10^6^**	NA	NA	NA
Br59	*A. lituratus*	M	Valparais	NA	**2.10 × 10^2^**	NA	NA	NA
A354	*A. planirostris*	M	Birigui	**1.09 ×10^5^**	**5.02 × 10^4^**	**4.73 × 10^5^**	**9.68 × 10^3^**	**6.01 × 10^3^**
Br61	*Carollia perspicillata*	F	Araçatuba	**4.73 × 10^4^**	**1.17 × 10^3^**	NA	NA	NA
Br62	*C. perspicillata*	F	Araçatuba	**1.99 × 10^7^**	**6.96 × 10^7^**	NA	NA	NA
Br63	*C. perspicillata*	F	Araçatuba	2.71 × 10^2^	8.61 × 10°	NA	NA	NA
Br65	*C. perspicillata*	M	Araçatuba	2.23 × 10^1^	2.88 × 10^2^	NA	NA	NA
Br68	*C. perspicillata*	F	Araçatuba	2.35 × 10^1^	Neg	NA	NA	NA
Br69	*C. perspicillata*	M	Araçatuba	**5.95 × 10^7^**	**1.99 × 10^6^**	NA	NA	NA
Br70	*C. perspicillata*	M	Araçatuba	**8.20 × 10^7^**	**1.85 × 10^5^**	NA	NA	NA
Br71	*C. perspicillata*	F	Araçatuba	5.37 × 10^3^	5.38 × 10^2^	NA	NA	NA
Br72	*C. perspicillata*	M	Araçatuba	4.70 × 10^2^	6.11 × 10^1^	NA	NA	NA
Br74	*C. perspicillata*	M	Araçatuba	**3.54 × 10^5^**	**8.84 × 10^6^**	NA	NA	NA
Br76	*C. perspicillata*	M	Araçatuba	**1.18 × 10^6^**	**1.52 × 10^7^**	NA	NA	NA
Br77	*C. perspicillata*	F	Araçatuba	Neg	1.81 × 10^2^	NA	NA	NA

*Numbers in bold are samples used in arenavirus isolation attempts. NA, tissue not available; Neg, negative †Samples were collected from *Artibeus lituratus* bats in forest areas of Valparaiso in 2007, *A. planirostris* bat in an urban area of Birigui in 2011, and *Carollia perspicillata* bats in forest areas of Araçatuba in 2007.

All arenavirus-positive animals appeared healthy, suggesting limited negative effects of arenavirus infection on bat hosts. This observation was similar in rodent arenavirus hosts (*10*) and consistent with high TCRV seroprevalence in a serologic survey (*4*) but different from experimental TCRV infections (*5*), likely because of different routes and high doses used for infecting bats in laboratory settings. High seroprevalence and low arenavirus detection rates suggest that arenaviruses do not infect bats persistently, which is distinct from results for rodent arenavirus infections (*11*). Lack of persistence is important for public health because it indicates potential limitations of arenavirus shedding by bat hosts whose lifespan is <8–12 years (*12*).

We detected arenavirus RNA in multiple organs at similar concentrations, including spleens (mean, 1.2 × 10^7^ RNA copies/mg) and lungs (mean, 6.4 × 10^6^ RNA copies/mg) (p = 0.53 by Mann-Whitney U test) (Table 2), suggesting systemic infection similar to that observed in experimentally infected bats (*5*). We observed the highest arenavirus RNA concentration in the single arenavirus-positive intestine specimen, followed by the spleen, lung, liver, and kidney in that animal (Table 2). High arenavirus RNA concentrations in intestines are consistent with virus shedding through the enteric route, which has been observed during experimental infections with TCRV (*5*). Although rodents shed arenaviruses primarily through urine and saliva, shedding also occurs in feces (*2*). Determining differences in arenavirus transmission routes between bats and rodents will require further investigation. We were unsuccessful isolating bat arenaviruses from organ homogenates despite repeated attempts (Appendix), likely because of tissue degradation under tropical conditions.

**Figure 2:**
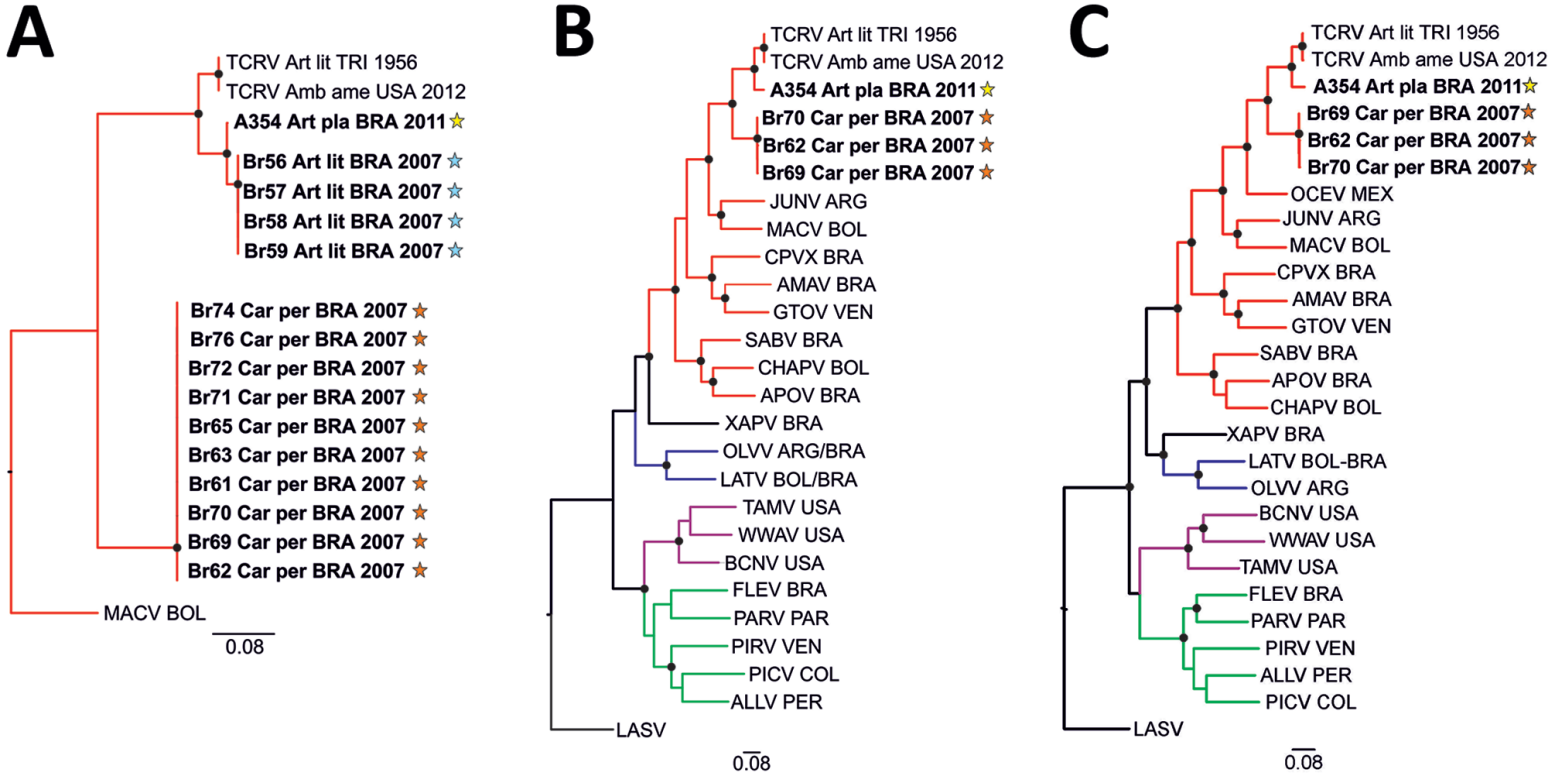
Phylogenetic analyses of highly diverse arenaviruses in neotropical bats, Brazil. Maximum-likelihood consensus trees compare partial RNA-dependent RNA polymerase genes (A), complete large (L) segment genes (B), and complete small (S) segment genes (C) from arenaviruses detected in *Artibeus* and *Carollia* spp. bats. Phylogenetic trees were generated using MEGA X software (https://www.megasoftware.net). Bold indicates sequences obtained from this study. Stars indicate regions where arenavirus-positive bat hosts were detected (Figure 1, panel B). Black dots at tree nodes represent bootstrap values >75% (1,000 replicates). Green lines indicate clade A new world arenaviruses, red lines indicate clade B new world arenaviruses, blue lines indicate clade C new world arenaviruses, and purple lines indicate recombinant new world arenaviruses (tentative clade D) (*14*). GenBank sequences used for comparisons and virus abbreviations (accession nos.): TCRV_Art_lit_TRI_1956, Tacaribe virus strain TRVL-11573 L segment (MT081317) and S segment (MT081316.1); TCRV_Amb_ame_USA_2012, Tacaribe virus Florida L segment (KF923401) and S segment (KF923400); OCEV, Ocozocoautla de Espinosa Virus S segment (JN897398; L segment not available); JUNV, Junin virus L segment (AY35802) and S segment (NC_005081.1); MACV, Machupo virus L segment (AY358021) and S segment (NC_005078.1); CPVX, Cupixi virus L segment (AY216519) and S segment (NC_010254.1); AMAV, Amapari virus L segment (AY216517) and S segment (NC_010247.1); GTOV, Guanarito virus L segment (AY358024) and S segment (NC_005077.1); SABV, Sabia virus L segment (AY358026) and S segment (NC_006317.1); APOV, Apore virus L segment (MF317491) and S segment (MF317490); CHAPV, Chapare virus L segment (EU260464) and S segment (EU260463.1); XAPV, Xapuri virus L segment (MG976577) and S segment (NC_055439.1), LATV, Latino virus L segment (EU627612) and S segment (NC_010758.1); OLVV, Oliveros virus L segment (AY216514) and S segment (NC_010248.1); BCNV, Bear Canyon virus L segment (AY924390) and S segment (NC_010256.1); WWAV, Whitewater Arroyo virus L segment (AY924395) and S segment (NC_010700.1); TAMV, Tamiami virus L segment (AY924393) and S segment (NC_010701.1); FLEV, Flexal virus L segment (EU627611) and S segment (NC_010757.1); PARV, Parana virus L segment (EU627613) and S segment (NC_010756.1); PIRV, Pirital virus L segment (AY494081) and S segment (NC_005894.1); PICV, Pichinde virus L segment (AF427517) and S segment (MK896487.1); LASV, Lassa virus L segment (U73034) and S segment (NC_004296.1); ALLV, Allpahuayo virus L segment (AY216502) and S segment (NC_010253.1). Origins of arenaviruses are indicated for each sample: ARG, Argentina; BOL, Bolivia; BRA, Brazil; COL, Colombia; PER, Peru; TRI, Trinidad; USA, United States of America; VEN, Venezuela. Scale bars indicate nucleotide substitutions per site.

We performed phylogenetic analysis of the partial sequence for the arenavirus RNA-dependent RNA polymerase gene obtained from RT-PCR screening. We found 2 NWA clades in bats from Brazil: 1 clade for both *Artibeus* spp. and 1 clade for *C. perspicillata* bats (GenBank accession nos. ON648806–16) (Figure 2, panel A). We obtained complete arenavirus coding sequences from 1 *A. planirostris* and 3 *C. perspicillata* bats (GenBank accession nos. ON648817–24) by using Illumina-based deep sequencing (Illumina, https://www.illumina.com); genome organization was identical to other mammarenaviruses. Both arenaviruses formed a well-supported monophyletic clade with TCRV in sister relationship to Junin and Machupo viruses (Figure 2, panel B) and Ocozocoautla de Espinosa virus that was possibly responsible for a hemorrhagic fever outbreak in Mexico (Figure 2, panel C) (*13*). These results highlight the genetic relationship of those bat-associated arenaviruses with highly pathogenic NWAs (Appendix Table 2). Identical topology in phylogenetic reconstructions argued against potential reassortment (Figure 2, panels B, C), and homogeneous sequence distances and recombination analyses along the genome did not indicate recombination events (Appendix Figure 2).

The *A. planirostris* bat was infected with a previously unknown TCRV strain (Appendix Table 2) that had an amino acid identity of 93.8%–95.5% with other TCRV sequences, depending on the protein analyzed. The arenaviruses from *C. perspicillata* bats formed a separate species in clade B of the TCRV serogroup (Figure 2, panels B, C). Species assignment relied on taxonomic criteria (*14*) that included exclusive detection in a distinct host, nucleotide sequence identity of <80% in the small segment, and 88.6%–90% amino acid identity in the nucleocapsid protein compared with TCRV and pairwise sequence comparison (https://www.ncbi.nlm.nih.gov/sutils/pasc/viridty.cgi?textpage=overview) results for large and small segments (Appendix Figure 3). The 5′ and 3′ ends of large and small genomic segments obtained from the newly identified arenavirus from *C. perspicillata* bats were nearly identical to TCRV, consistent with a close genetic relationship between those NWAs (Appendix Table 3, Figure 4). We propose that the arenavirus sequenced from *C. perspicillata* bats should be named Tietê virus (species *Tiête mammarenavirus*) and abbreviated as TETV; the name comes from the main river located <4 km from the capture site (Figure 1).

## Conclusions

Arenavirus genetic diversity is hypothesized to result from a complex macro-evolutionary pattern that includes both co-evolution and host switching in the Muridae family of rodents. In South America, arenaviruses might have co-evolved with rodents in the Sigmodontinae subfamily, with the exception of TCRV (*10*). Further investigation will be required to determine whether bat arenaviruses evolved from an ancestral host switch involving rodents, which would be consistent with the genetic relationship between TCRV or Tietê virus and rodent-derived Ocozocoautla de Espinosa virus, or whether bats and arenaviruses co-evolved. Of note, bats play an essential role in ecosystems, and stigmatization of bats as sources of zoonotic viruses is unwarranted.

In summary, the epidemiology, genealogy, and zoonotic potential of bat arenaviruses deserve further investigation. Our results suggest that bats are an underrecognized arenavirus reservoir. 

Appendix Additional information for highly diverse arenaviruses in neotropical bats, Brazil.

## References

[1] Guth S, Mollentze N, Renault K, Streicker DG, Visher E, Boots M, et al. Bats host the most virulent-but not the most dangerous-zoonotic viruses. Proc Natl Acad Sci U S A. 2022;119:e2113628119. 10.1073/pnas.2113628119 35349342 PMC9168486

[2] Charrel RN, de Lamballerie X. Zoonotic aspects of arenavirus infections. Vet Microbiol. 2010;140:213–20. 10.1016/j.vetmic.2009.08.027 19748747

[3] Downs WG, Anderson CR, Spence L, Aitken THG, Greenhall AH. Tacaribe virus, a new agent isolated from Artibeus bats and mosquitoes in Trinidad, West Indies. Am J Trop Med Hyg. 1963;12:640–6. 10.4269/ajtmh.1963.12.640 22324073

[4] Malmlov A, Seetahal J, Carrington C, Ramkisson V, Foster J, Miazgowicz KL, et al. Serological evidence of arenavirus circulation among fruit bats in Trinidad. PLoS One. 2017;12:e0185308. 10.1371/journal.pone.0185308 28953976 PMC5617188

[5] Cogswell-Hawkinson A, Bowen R, James S, Gardiner D, Calisher CH, Adams R, et al. Tacaribe virus causes fatal infection of an ostensible reservoir host, the Jamaican fruit bat. J Virol. 2012;86:5791–9. 10.1128/JVI.00201-12 22379103 PMC3347293

[6] Sayler KA, Barbet AF, Chamberlain C, Clapp WL, Alleman R, Loeb JC, et al. Isolation of Tacaribe virus, a Caribbean arenavirus, from host-seeking *Amblyomma americanum* ticks in Florida. PLoS One. 2014;9:e115769. 10.1371/journal.pone.0115769 25536075 PMC4275251

[7] Moreno H, Möller R, Fedeli C, Gerold G, Kunz S. Comparison of the innate immune responses to pathogenic and nonpathogenic clade B New World arenaviruses. J Virol. 2019;93:e00148–19. 10.1128/JVI.00148-19 31270228 PMC6744228

[8] Holzerland J, Leske A, Fénéant L, Garcin D, Kolakofsky D, Groseth A. Complete genome sequence of Tacaribe virus. Arch Virol. 2020;165:1899–903. 10.1007/s00705-020-04681-9 32462284 PMC7351806

[9] Vieth S, Drosten C, Lenz O, Vincent M, Omilabu S, Hass M, et al. RT-PCR assay for detection of Lassa virus and related Old World arenaviruses targeting the L gene. Trans R Soc Trop Med Hyg. 2007;101:1253–64. 10.1016/j.trstmh.2005.03.018 17905372

[10] Grande-Pérez A, Martin V, Moreno H, de la Torre JC. Arenavirus quasispecies and their biological implications. Curr Top Microbiol Immunol. 2016;392:231–76. 10.1007/82_2015_468 26472215 PMC7122398

[11] Hoffmann C, Wurr S, Pallasch E, Bockholt S, Rieger T, Günther S, et al. Experimental Morogoro virus infection in its natural host, *Mastomys natalensis.* Viruses. 2021;13:851. 10.3390/v13050851 34067011 PMC8151005

[12] Jones ML. Longevity of captive mammals. Zool Garten N. F. Jena. 1982;52;113–28 [cited 2022 Nov 10]. http://www.rhinoresourcecenter.com/pdf_files/125/1256468598.pdf

[13] Cajimat MNB, Milazzo ML, Bradley RD, Fulhorst CF. Ocozocoautla de espinosa virus and hemorrhagic fever, Mexico. Emerg Infect Dis. 2012;18:401–5. 10.3201/eid1803.111602 22377271 PMC3309595

[14] Radoshitzky SR, Bào Y, Buchmeier MJ, Charrel RN, Clawson AN, Clegg CS, et al. Past, present, and future of arenavirus taxonomy. Arch Virol. 2015;160:1851–74. 10.1007/s00705-015-2418-y 25935216

