# Supplementary material for: Highly Diverse Arenaviruses in Neotropical Bats, Brazil
Source: Emerg Infect Dis. 2022 Dec;28(12):2528–33. doi: 10.3201/eid2812.220980 (PMC9707603; doi:10.3201/eid2812.220980)
Supplement: Appendix — Additional information for highly diverse arenaviruses in neotropical bats, Brazil. [file 22-0980-Techapp-s1.pdf]

Article DOI: https://doi.org/10.3201/eid2812.220980

Highly Diverse Arenaviruses in Neotropical Bats, Brazil

Appendix

Additional Methods

Bat collection

Bats were collected from 36 sites during 2007–2015 that included pristine forest areas, forest fragments, and urban environments from 4 main geographic regions: National Park of Iguaçu, (south of Parana state characterized as a pristine area), central region of Parana state from 2 forest fragments, northwest region of São Paulo state (urban areas and forest fragments), and southwest region of São Paulo state (metropolitan areas and few urban forest fragments) (Figure 1). Bat species were identified by field biologists based on morphological criteria as described previously (*1*).

New World Arenavirus Screening Assay

We performed arenavirus screening by using broadly reactive primers (*2*) slightly modified according to alignments of New World arenavirus genomes from GenBank (Appendix Figure 2). Final primer sequences were: LVL_3359A_Mod, 5′-AGRCTTAGTGWGAGRGARAGCAAYTC-3′ and LVL_3754D_Mod, 5′-CWCATDATTGGICCCCAYTTWGARTGRTC-3′. We extracted viral RNA from 30 mg of bat tissue by using the MagNA Pure 96 DNA and Viral NA Large Volume Kit (Roche, https://www.roche.com). We screened samples by using SuperScript III One-Step RT-PCR System with Platinum Taq DNA Polymerase (Thermo Fisher Scientific, https://www.thermofisher.com). We performed PCR following the manufacturer's instructions in a final volume of 25 µL containing 5 µL RNA, 0.6 µmol/L primers, 12.5 µL 2× reaction mix buffer, 0.8 mmol/L $MgSO_4$, 1 µg bovine serum albumin, and 1 µL SuperScript III/Platinum Taq polymerase mix. Amplification was performed as follows: 50°C for 30 min followed by 95°C for

3 min; 45 cycles of 94°C for 15 s, 56°C for 20 s, and 72°C for 35 s; and final elongation at 72°C for 5 min. The primers generated an amplicon of 395 bp (*2*).

Virus Quantification

We quantified viruses by using one-step real-time RT-PCR with specific primers and probes for *Artibeus* sp. and *Carollia* sp. arenaviruses targeting the L gene: Art_Arena_Fwd, 5′-AACTGCCAACTGCCTCTGCA-3′; Art_Arena_Rev, 5′-CAAGGAGCAAGTGGGTTCCA-3′; Art_Arena_Probe, 5′-TGTTGAGATCCCCAACGTAAAGTTCCCT-3′; Car_Arena_Fwd, 5′-TTAGGTCCCCAACATACAGTTC-3′; Car_Arena_Rev, 5′-CACTAGCATCAGTTCAAATAATGG-3′; Car_Arena_Probe, 5′-AACCCACCTGTTCCTTATAACTCAAGCC-3′. We performed RT-PCR by using 25 µL reaction volumes containing 5 µL RNA, 2.0 mmol/L $MgCl_2$, 0.2 mmole/L (each) dNTPs, 0.4 µmol/L of each primer, 0.3 µmol/L probe, 1× PCR buffer, and 1 µL SuperScript III/Platinum Taq polymerase. We amplified the cDNA as follows: 50°C for 20 min, followed by 98°C for 3 min; 45 cycles of 98°C for 15 s, 58°C for 30 s (fluorescence was read at the 58°C step); and cooling at 40°C for 30 s. We quantified arenavirus RNA according to photometrically quantified cRNA transcribed from a gBlock synthetic gene fragment (Integrated DNA Technologies, Inc., https://www.idtdna.com) by using the MEGAscript T7 transcription kit (Thermo Fisher Scientific).

Virus Isolation Attempts

We attempted to isolate arenaviruses from arenavirus-positive organs of *Artibeus* sp. and *Carollia* sp. bats by using the cell lines Vero E6, C6/36, BHK-21, and CarLu/1 (lung epithelial cells isolated from a *Carollia perspicillata* bat) (*3*) and the immortalized primary cell line AJL derived from *Artibeus jamaicensis* lung tissue (*4*,*5*). Briefly, we shredded and homogenized 25 mg of arenavirus-positive tissue in 500 µL sterile 1× phosphate buffered saline at 30 Hz for 3 min by using a TissueLyser (QIAGEN, https://www.qiagen.com). To avoid cytotoxicity, we diluted the homogenates 5× and 50× in Leibovitz's L-15 Medium (C6/36 cells) or Dulbecco's Modified Eagle Medium (remaining cell lines) (Thermo Fisher, https://www.thermofisher.com) in 24-well plates 1 d before adding to cells. Cells were seeded at 1.5×10^5 (Vero cells), 2.5×10^5 (C6/36), 0.9×10^5 (BHK-21), 1.4×10^5 (CarLu), and 1.5×10^5 (AJL) cells/well. We prepared the cells in medium supplemented with 1% fetal bovine serum (Thermo Fisher Scientific) 1 h before

infection. After 1 h, we removed all medium and added a total of 200 µL homogenate to each well in a 24-well plate. The cells were incubated for 1 h at 37°C in 5% CO_2, except for C6/36, which were incubated for 1 h at 28°C without CO_2. We recovered the inoculum (200 µL) and stored for further experiments. We added 500 µL of fresh Leibovitz's L-15 Medium containing 5% fetal bovine serum, 1% penicillin-streptomycin, and 1% of 200 mM L-glutamine to the C6/36 cells and Dulbecco's Modified Eagle Medium supplemented with 10% fetal bovine serum, 1% penicillin-streptomycin, and 1% nonessential amino acids to the other cells (Thermo Fisher Scientific). We observed the inoculated cells every 24 h to monitor cytopathic effects. We performed 5 serial blind passages every 5 d and collected supernatant samples (100 µL); cells were discarded if real-time RT-PCR results were negative for arenavirus.

Statistics

Confidence intervals were calculated by using Prism software (GraphPad, https://www.graphpad.com). We performed Mann-Whitney U tests using SPSS software, version 13.0 (IBM, https://www.ibm.com).

References

1. Góes LGB, Campos ACA, Carvalho C, Ambar G, Queiroz LH, Cruz-Neto AP, et al. Genetic diversity of bats coronaviruses in the Atlantic Forest hotspot biome, Brazil. Infect Genet Evol. 2016;44:510–3. PubMed https://doi.org/10.1016/j.meegid.2016.07.034

2. Vieth S, Drosten C, Lenz O, Vincent M, Omilabu S, Hass M, et al. RT-PCR assay for detection of Lassa virus and related Old World arenaviruses targeting the L gene. Trans R Soc Trop Med Hyg. 2007;101:1253–64. PubMed https://doi.org/10.1016/j.trstmh.2005.03.018

3. Kopp A, Gillespie TR, Hobelsberger D, Estrada A, Harper JM, Miller RA, et al. Provenance and geographic spread of St. Louis encephalitis virus. mBio. 2013;4:e00322-13. PubMed https://doi.org/10.1128/mBio.00322-13

4. Moreira-Soto A, Soto-Garita C, Corrales-Aguilar E. Neotropical primary bat cell lines show restricted dengue virus replication. Comp Immunol Microbiol Infect Dis. 2017;50:101–5. PubMed https://doi.org/10.1016/j.cimid.2016.12.004

5. Moreira-Soto A, Taylor-Castillo L, Vargas-Vargas N, Rodríguez-Herrera B, Jiménez C, Corrales-Aguilar E. Neotropical bats from Costa Rica harbour diverse coronaviruses. Zoonoses Public Health. 2015;62:501–5. PubMed https://doi.org/10.1111/zph.12181

Appendix Table 1. Bat species and tissues screened for arenavirus RNA in study of highly diverse arenaviruses in neotropical bats, Brazil*

Bat species	No. intestine	No. (%) positive	No. spleens	No. (%) positive	No. livers	No. (%) positive	No. lung	No. (%) positive	Total no. specimens
Artibeus fimbriatus	3	0	0	0	3	0	0	0	6
A. lituratus	145	0	100	2 (2.0)	95	0	64	4 (6.25)	404
A. obscurus	2	0	2	0	2	0	2	0	8
A. planirostris	8	1 (12.5)	8	1 (12.5)	9	1 (11.1)	7	1 (14.29)	32
Carollia perspicillata	45	0	40	11 (27.5)	31	0	34	12 (35.3)	150
Chrotopterus auritus	0	0	0	0	1	0	0	0	1
Cynomops planirostris	11	0	11	0	11	0	11	0	44
Desmodus rotundus	56	0	57	0	61	0	68	0	242
Eptesicus furinalis	17	0	14	0	17	0	17	0	65
Eumops auripendulus	2	0	2	0	2	0	2	0	8
E. glaucinus	103	0	103	0	102	0	106	0	414
E. perotis	12	0	12	0	12	0	12	0	48
Glossophaga soricina	66	0	66	0	67	0	70	0	269
Lasiurus blossevillii	2	0	2	0	2	0	2	0	8
L. cinereus	1	0	1	0	1	0	1	0	4
L. ega	2	0	2	0	2	0	2	0	8
Molossops neglectus	1	0	1	0	1	0	1	0	4
M. temmincki	2	0	2	0	2	0	2	0	8
Molossus molossus	239	0	227	0	238	0	239	0	943
M. rufus	145	0	153	0	148	0	160	0	606
Myotis nigricans	33	0	28	0	35	0	33	0	129
M. riparius	0	0	0	0	1	0	0	0	1
Noctilio albiventris	0	0	0	0	0	0	2	0	2
Nyctinomops laticaudatus	4	0	3	0	3	0	4	0	14
N. macrotis	1	0	1	0	1	0	1	0	4
Phyllostomus discolor	2	0	2	0	2	0	2	0	8
Platyrrhinus lineatus	6	0	6	0	6	0	6	0	24
Promops nasutus	1	0	1	0	1	0	1	0	4
Pygoderma bilabiatum	1	0	1	0	1	0	1	0	4
Sturnira lilium	29	0	15	0	23	0	5	0	72
Tadarida brasiliensis	30	0	29	0	30	0	30	0	119
Vampyressa pusila	0	0	0	0	1	0	0	0	1
Unknown	4	0	4	0	4	0	4	0	16
Total	973	1 (0.1)	893	15 (1.7)	915	1 (0.1)	889	17 (1.9)	3670

*Species in bold font had samples that were positive for arenavirus RNA.

Appendix Table 2. Comparisons of nucleotide and amino acid sequence distances of arenaviruses detected in *Artibeus planirostris* (sample A354) and *Carollia perspicillata* (sample Br70) bats in study of highly diverse arenaviruses in neotropical bats, Brazil*

		L segment			S segment		
Sample	Arenavirus†	L segment (nt)	ZP (aa)	RdRp (aa)	S segment (nt)	GPC (aa)	NP (aa)
A354	TCRV TRVL	0.09	0.06	0.04	0.08	0.05	0.04
	TCRV USA	0.09	0.06	0.04	0.09	0.05	0.04
	OCEV	NA	NA	NA	0.27	0.25	0.17
	JUNV	0.33	0.29	0.29	0.30	0.32	0.20
	MACV	0.32	0.28	0.29	0.31	0.35	0.18
	Br70	0.23	0.17	0.15	0.21	0.13	0.10
Br70	TCRV TRVL	0.23	0.18	0.14	0.21	0.12	0.10
	TCRV USA	0.23	0.18	0.14	0.21	0.13	0.10
	OCEV	NA	NA	NA	0.26	0.25	0.16
	JUNV	0.33	0.28	0.30	0.30	0.29	0.20
	MACV	0.32	0.27	0.29	0.31	0.33	0.17
	A354	0.23	0.17	0.15	0.21	0.13	0.10

*Genetic distances were calculated by using MEGA X (MEGA, https://www.megasoftware.net). aa, amino acid; GPC, glycoprotein precursor; NA, sequence not available; NP, nucleocapsid protein; nt, nucleotide; RdRp, RNA-dependent RNA polymerase; ZP, zinc binding protein.
†GenBank sequences used for comparisons: TCRV TRVL, Tacaribe virus, strain TRVL-11573 L segment (MT081317) and S segment (MT081316.1); TCRV USA, Tacaribe virus, Florida isolate L segment (KF923401) and S segment (KF923400); OCEV, Ocozocoautla de Espinosa virus S segment (JN897398.1); JUNV, Junin virus L segment (AY358022) and S segment (NC_005081.1); and MACV, Machupo virus L segment (AY358021) and S segment (NC_005078.1).

Appendix Table 3. Primers used to determine sequences of 5′ and 3′ ends of genomic segments L and S after rapid amplification of cDNA ends (RACE) in study of highly diverse arenaviruses in neotropical bats, Brazil*

Primer name	Primer sequence	Target segment
5′_RACE_A354_S_R3	CTCTTGCATGAAACTAATAAACTGC	S
5′_RACE_A354_S_R2	ATGCAATGTTGAGTGCTTCCTG	S
5′_RACE_A354_S_R1	TACTATGCAGATGAGGCTGACT	S
3`_RACE_A354_S_F1	AACGTCTGACTTGACTGTTTGG	S
3`_RACE_A354_S_F2	GAACTGACTCAATCCTTTTCTAAG	S
3`_RACE_A354_S_F3	GGCACTTCCTTGGATTGAGC	S
5′_RACE_A354_L_R3	TTCTGAGTTCGATTGCAGTTGC	L
5′_RACE_A354_L_R2	TGTGGTGGCTTCTCGAGATC	L
5′_RACE_A354_L_R1	CTTCTGAATTCTGCTGCTTGTG	L
3′_RACE_A354_L_F1	AAACCTTCTTGGAGTAATGATCCT	L
3′_RACE_A354_L_F2	CTGATGGGCAAACTCATGCC	L
3′_RACE_A354_L_F3	CAGGTCTTTGAGTTCAGAAACAG	L
5′_RACE_Car_S_R1	CATCTGTATAGGTTTACCAGACC	S
5′_RACE_Car_S_R2	TACAAGTGCGACATTGAGTGCT	S
5′_RACE_Car_S_R3	CTGCATGAAACTGATGAACTGC	S
3`_RACE_Car_S_F1	CATCTTTGAGAATATCTGACTTGAC	S
3`_RACE_Car_S_F2	GACTCAGCCCTTTCCTTAGG	S
3`_RACE_Car_S_F3	AAGCTGGGCACCTCTTTGGA	S
5′_RACE_Car_L_R1	CAACAGCACTTGCAGTTATAACG	L
5′_RACE_Car_L_R2	CTGCCACTCTTCTGAACTCC	L
5′_RACE_Car_L_R3	AGAGTAGAACAGACCTTTGAGC	L
3′_RACE_Car_L_F1	TAGGAGCTTGAAACCTTCTTGG	L
3′_RACE_Car_L_F2	TGAGTGACATTGTGACAAGAAGG	L
3′_RACE_Car_L_F3	TGTCTATTTGGAATGTGTTTCCTG	L

*Rapid amplification of cDNA ends was performed by using the 5′/3′ RACE Kit (Roche, https://www.roche.com) in accordance with the manufacturer's instructions.

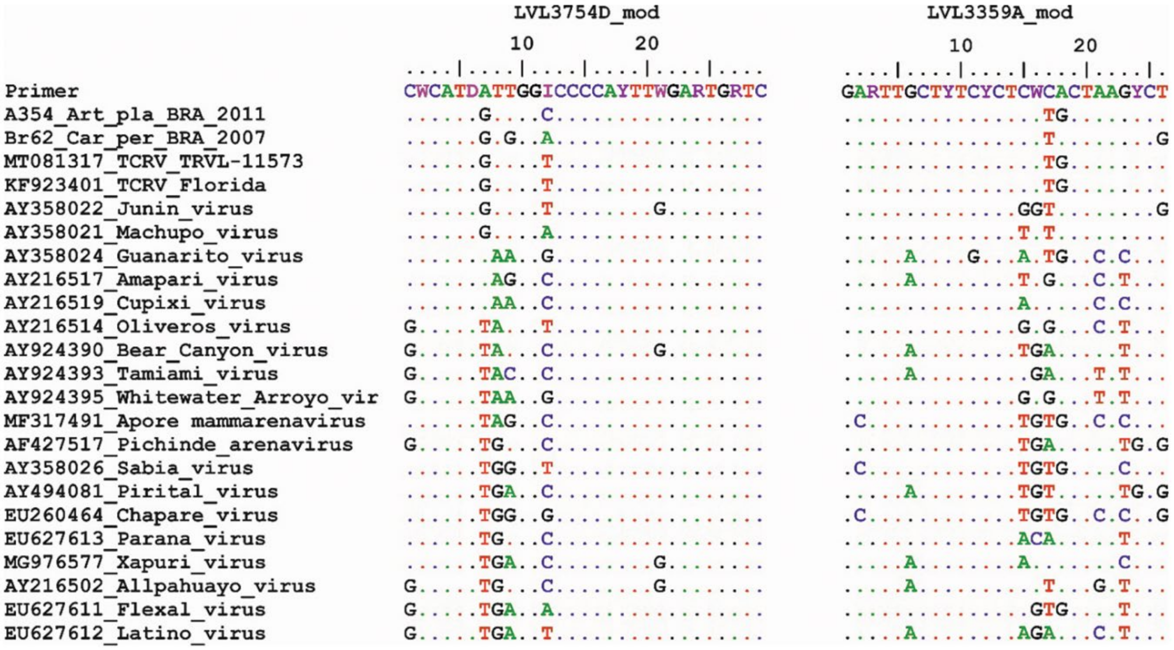

Appendix Figure 1. Comparison of binding sites of primers used for arenavirus screening between 23 L segment gene sequences from New World arenaviruses in study of highly diverse arenaviruses in neotropical bats, Brazil. The binding sites of primers LVL3754D_mod (5′–3′) and LVL 3359A_mod (3′–5′ genome binding site of primer is shown) were used as references. Nucleotide differences are indicated by letters; identical bases are shown as dots.

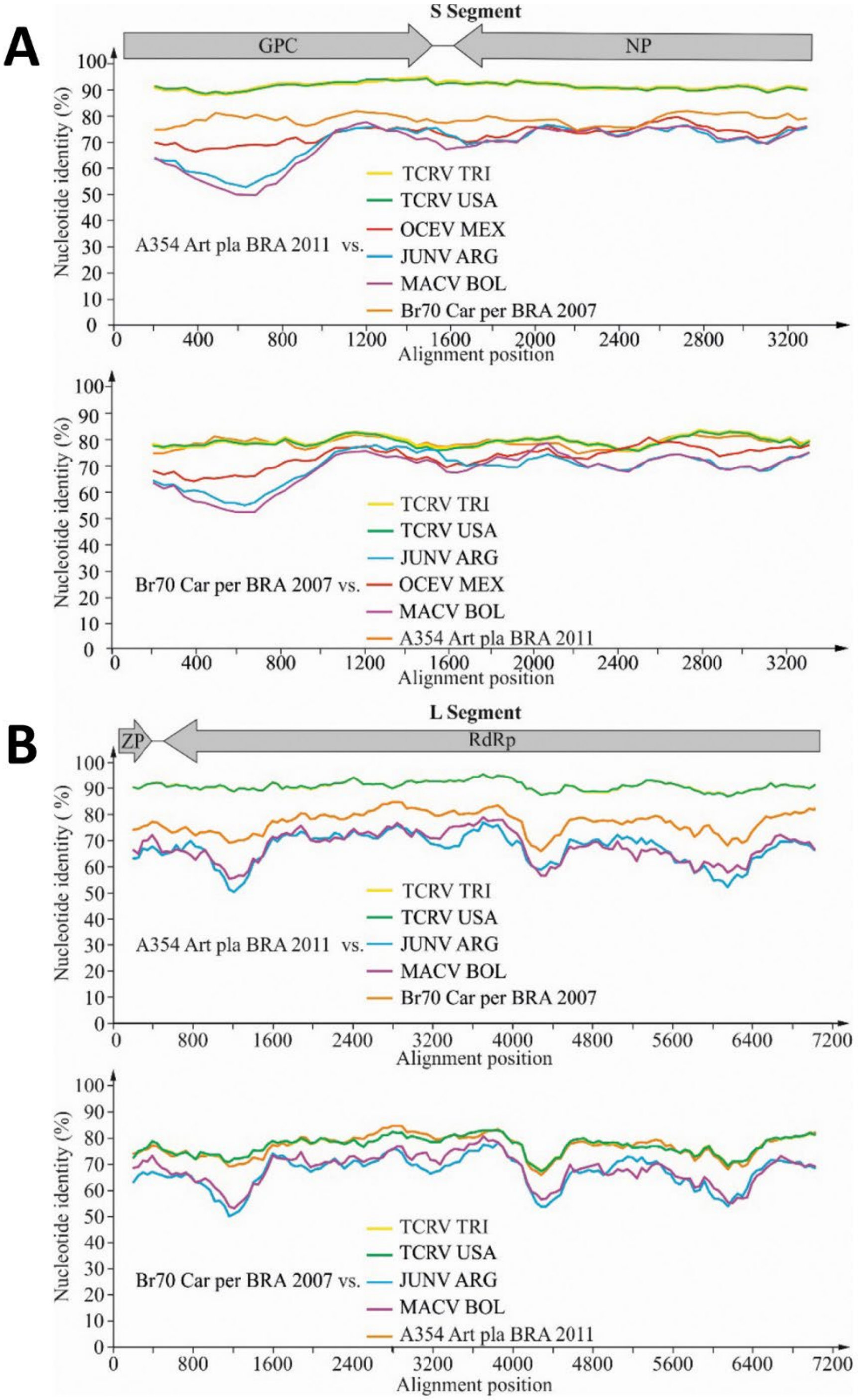

Appendix Figure 2. Genetic similarities between L and S segments of bat arenaviruses in study of highly diverse arenaviruses in neotropical bats, Brazil. We used SSE v1.4 software (http://www.virus-evolution.org/Downloads/Software) to generate genetic similarity plots to compare arenaviruses from *Artibeus planirostris* (sample A354) and *Carollia perspicillata* (sample Br70) bats with GenBank sequences for: TCRV_TRI, Tacaribe virus TRVL-11573 L segment (accession no. MT081317) and S segment (MT081316.1); TCRV USA, Tacaribe virus isolate Florida L segment (KF923401) and S

segment (KF923400); OCEV, Ocozocoautla de Espinosa virus S segment (JN897398.1) (L segment not available); JUNV, Junin virus L segment (AY358022) and S segment (NC_005081.1); and MACV, Machupo virus L segment (AY358021) and S segment (NC_005078.1). We used a 400 nt window with a 50 nt step (window was moved 50 nt in succession) and the p-distance model in SSE V1.4 software (http://www.virus-evolution.org/Downloads/software). Arenavirus genome organization includes 2 ambisense single-stranded RNA segments: a large (L) segment encoding RNA-dependent RNA polymerase (RdRp) and zinc binding protein (ZP) and a small (S) segment encoding the nucleocapsid protein (NP) and glycoprotein precursor (GPC). Recombination analyses were performed using the RDP4 program (http://web.cbio.uct.ac.za/~darren/rdp.html) and did not indicate recombinant events had occurred.

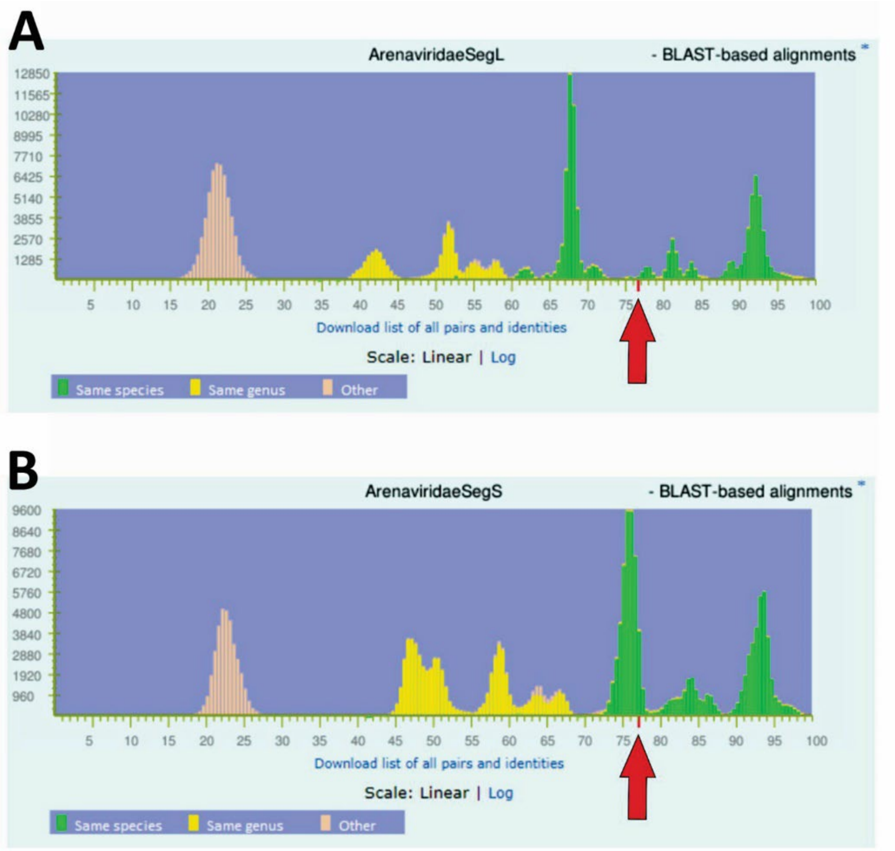

Appendix Figure 3. Sequence comparisons in study of highly diverse arenaviruses in neotropical bats, Brazil. We used the PAirwise Sequence Comparison (PASC) tool (https://www.ncbi.nlm.nih.gov/sutils/pasc/viridty.cgi?textpage=overview) to compare the A) L segment and B) S segment of the proposed new species *Tiête mammarenavirus* (sample Br70) with other members of the Arenaviridae family. Graphs already exist online for both gene segments in this virus family. The graphs show the percentage sequence identity of different family members on the x-axis and genome entries on the y-axis. We added our sequence to the program, and the red arrow indicates the percentage sequence identity of our sample with other viruses within the Arenaviridae family.

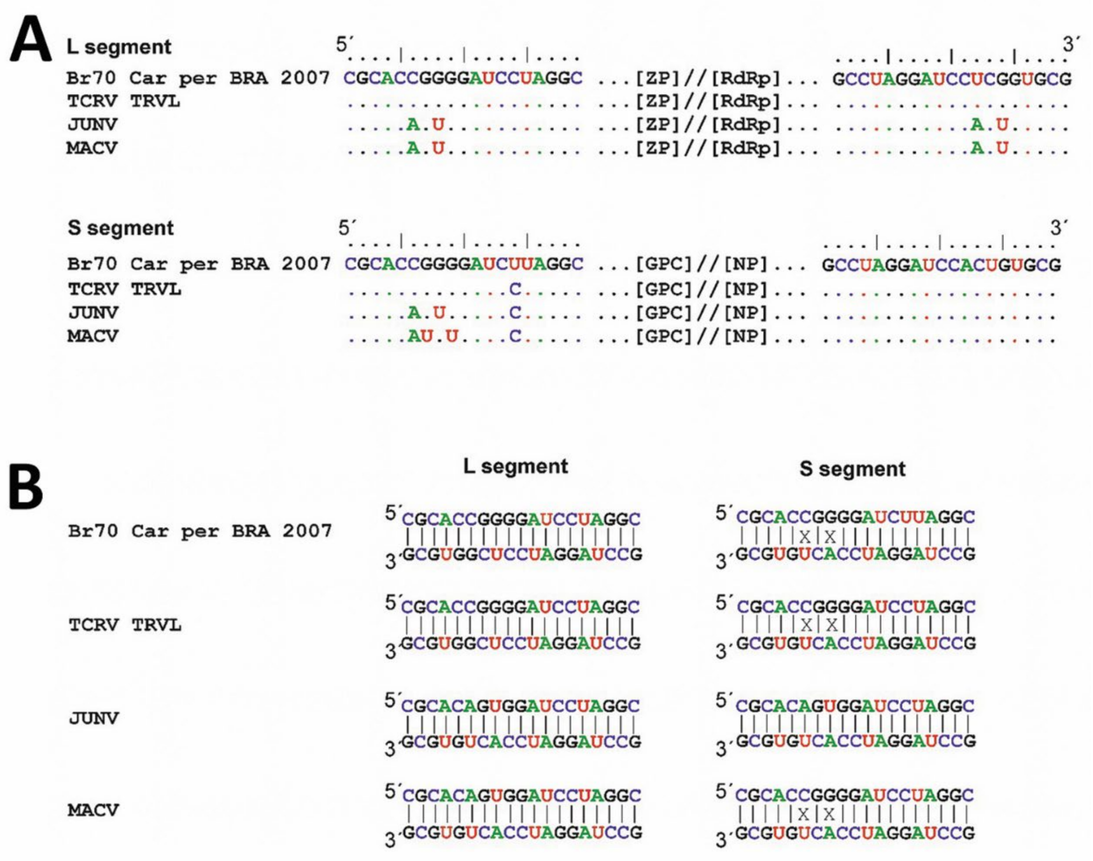

Appendix Figure 4. Analysis of genome termini of arenaviruses in study of highly diverse arenaviruses in neotropical bats, Brazil. We compared sequences of the 5′- and 3′-ends of L and S genomic segments obtained from the newly identified arenavirus (*Tiête mammarenavirus*) from *Carollia perspicillata* (specimen Br70 Car per BRA 2007) with those of other arenaviruses. A) Terminal sequences from Br70 Car per BRA 2007 were compared with the following GenBank reference sequences: TCRV-TRVL, Tacaribe virus TRVL-11573 L segment (accession no. MT081317) and S segment (MT081316.1); JUNV, Junin virus L segment (AY358022) and S segment (NC_005081.1), and MACV, Machupo virus L segment (AY358021) and S segment (KM198592.1). Identical bases are shown by dots. B) Complementarity was evaluated between the terminal 19 nts in the 5′- and 3′-untranslated regions of L and S segments. Mismatches are indicated by X, and base pairings by vertical lines. GPC, glycoprotein precursor; L, large segment; NP, nucleocapsid protein; RdRp, RNA-dependent RNA polymerase; S, small segment; ZP, zinc binding protein.
